# Gut microbiota dysbiosis in diabetic nephropathy: mechanisms and therapeutic targeting via the gut-kidney axis

**DOI:** 10.3389/fendo.2025.1661037

**Published:** 2025-09-18

**Authors:** Haiyan Jiang, Xiaoran Wang, Wei Zhou, Zhili Huang, Wen Zhang

**Affiliations:** ^1^ The First Affiliated Hospital of Zhejiang Chinese Medical University (Zhejiang Provincial Hospital of Chinese Medicine), Hangzhou, China; ^2^ Zhejiang Key Laboratory of Research and Translation for Kidney Deficiency-Stasis-Turbidity Disease, Zhejiang-Macau International Joint Laboratory of Integrated Traditional Chinese and Western Medicine for Nephrology and Immunology, Hangzhou, China; ^3^ Department of Nephrology, The First People’s Hospital of Hangzhou Lin’an District, Hangzhou, China; ^4^ Department of Hematology and Rheumatology, Tianchang People’s Hospital, Chuzhou, Anhui, China

**Keywords:** diabetic kidney disease, gut microbiota, gut-kidney axis, metabolism–immunity–barrier interaction, microbiome-targeted therapy

## Abstract

Diabetic kidney disease (DKD) is the primary microvascular complication of diabetes and a leading cause of chronic kidney disease (CKD) and end-stage renal disease (ESRD) worldwide, with its prevalence on the rise. Recent evidence has highlighted the crucial involvement of gut microbiota (GM) dysbiosis in the pathogenesis and progression of DKD, mediated through the gut-kidney axis. At the core of this process is a dynamic network involving metabolic, immune, and barrier dysfunction. Renal impairment—such as that seen in uremia—disrupts gut microbial composition and metabolic function. In turn, dysbiosis compromises intestinal barrier integrity, resulting in increased exposure to endotoxins and a reduction in the production of beneficial metabolites, notably short-chain fatty acids (SCFAs). This triad manifests as: (1) impaired metabolism, marked by decreased SCFAs (e.g., acetate), which weaken anti-inflammatory and immunomodulatory effects, alongside an accumulation of uremic toxins like trimethylamine N-oxide (TMAO) that trigger inflammatory pathways and renal fibrosis; (2) immune dysregulation, where increased endotoxin translocation (e.g., lipopolysaccharide, LPS) provokes systemic inflammation, oxidative stress, and immune cell infiltration (such as macrophages), contributing to renal inflammatory and fibrotic responses; and (3) barrier dysfunction, in which compromised intestinal barrier accelerates the translocation of detrimental microbial components, perpetuating a vicious cycle that exacerbates glomerulosclerosis, tubular injury, and renal function decline.Collectively, metabolic, immune, and barrier alterations reinforce one another and drive DKD progression via gut-derived metabolites and immune activation. Targeted interventions aiming to modulate the GM—using probiotics, prebiotics, or synbiotics—show promise in improving metabolic profiles, restoring gut barrier function, and mitigating DKD phenotypes. This review systematically elucidates the metabolism–immunity–barrier mechanisms by which GM dysbiosis contributes to DKD and discusses the translational potential of microbiome-targeted therapies. Further studies are needed to validate these findings and assess their long-term clinical efficacy.

## 1 Introduction

DKD, a major microvascular complication of diabetes, remains the leading cause of ESRD globally (1, 2). Its high prevalence not only necessitates long-term renal replacement therapy but also markedly increases the incidence of cardiovascular events in affected individuals (2, 3). In recent years, mounting evidence has highlighted the pivotal role of the gut–kidney axis in the pathogenesis and progression of DKD. Specifically, GM dysbiosis contributes to DKD evolution through metabolic disturbances, immune dysregulation, and barrier dysfunction:

### 1.1 Metabolic dimension

DKD is associated with a distinct gut microbial profile, notably a decreased abundance of butyrate-producing bacteria, which leads to reduced synthesis of beneficial SCFAs (4–6). Simultaneously, the accumulation of uremic toxins, such as indoxyl sulfate, exacerbates renal injury (2, 7, 8). These changes drive systemic activation of the renin–angiotensin system (RAS) and foster renal fibrosis (2, 7, 9).

### 1.2 Immune dimension

SCFA deficiency impairs their anti-inflammatory effects and the inhibition of histone deacetylases (HDACs) (4, 6, 10). Meanwhile, accumulation of microbial toxins acts synergistically with hyperactivation of the TLR4/NF-κB pathway (9, 11), leading to chronic systemic inflammation and oxidative stress that accelerate renal damage.

### 1.3 Barrier dimension

Dysbiosis impairs the integrity of intestinal epithelial tight junctions—such as zonula occludens-1 (ZO-1)—facilitating translocation of endotoxins into the bloodstream (8, 12). This establishes a deleterious “leaky gut–renal injury” cycle, which is further aggravated in DKD by hyperglycemia-induced depletion of the intestinal mucous layer, setting DKD apart from non-diabetic kidney diseases (NDKD) (5, 12, 13).

Compared with NDKD, the gut–kidney axis in DKD exhibits unique metabolic features: chronic hyperglycemia selectively suppresses butyrate metabolic pathways (6, 14), and the synergy between advanced glycation end products (AGEs)-RAGE signaling and gut-derived toxins accelerates the development of renal fibrosis (2, 7). In this review, we systematically explore the mechanisms whereby the interplay of metabolism, immunity, and barrier dysfunction propels DKD progression to ESRD. Additionally, we critically appraise the translational potential of microbiota-targeted interventions, such as dietary fiber supplementation to enhance SCFAs–GPR43 signaling, and probiotic modulation of Akkermansia to promote barrier restoration.

## 2 Clinical evidence linking gm dysbiosis to diabetic nephropathy

### 2.1 Characteristics of GM in patients with DN

#### 2.1.1 Alterations in microbial composition

Marked reduction in beneficial bacteria: Patients with DN exhibit a distinct gut microbial profile characterized by a significant decrease in SCFA-producing genera, such as *Akkermansia*, *Roseburia*, and *Alistipes*. Notably, a reduction in *Akkermansia* abundance is associated with the progression of renal fibrosis (p < 0.05), while decreased *Alistipes* correlates with heightened systemic inflammatory responses (p < 0.05) (15). These changes may contribute to compromised intestinal barrier function and immune dysregulation, thereby accelerating the pathological progression of nephropathy.

Enrichment of pathogenic bacteria: Conversely, the abundance of pathogenic bacteria, particularly *Escherichia-Shigella*, is increased in DN patients. These bacteria secrete endotoxins, such as LPS, which activate proinflammatory pathways in renal tissue (notably the NF-κB signaling pathway), thereby promoting glomerulosclerosis and tubular injury. Overall, gut dysbiosis exacerbates systemic inflammation and renal fibrosis in DN (16, 17).

Several studies have demonstrated a robust association between changes in microbial composition and renal function parameters. For instance, reduced *Roseburia* abundance is linked to an increased risk of DN (P = 0.00118; OR = 0.513, 95% CI: 0.343–0.768), suggesting a negative relationship with eGFR decline (18). Similarly, lower *Dialister* abundance is inversely correlated with DN risk (OR = 0.513, P = 0.00118) (18), further substantiating the detrimental impact of microbial dysbiosis on renal function. Moreover, Mendelian randomization (MR) analyses have confirmed a direct, negative causal effect of decreased SCFA-producing bacteria on DN progression (P < 0.05) (19), associating gut microbial alterations with renal function deterioration.

#### 2.1.2 Potential as biomarkers

Characteristic microbial profiles hold significant promise for distinguishing DN from NDKD. For instance, reduced abundance of Verrucomicrobia in DN patients serves as a phylum-level discriminator (18, 20). Notably, species-level biomarkers within the Prevotella genus exhibit strong clinical relevance. Prevotella copri is markedly enriched in diabetic patients with poor glycemic control (HbA1c >7.0%) and inversely correlates with healthy dietary patterns (e.g., fish-vegetable intake) ^21^. Its diagnostic power is robust, with an AUC of 0.93 (95% CI: 0.88–0.98) for distinguishing DN from non-DKD subjects via qPCR validation (21).

Additionally, Prevotella_9 demonstrates significant alterations in DKD. Metagenomic analyses reveal that Prevotella_9 species (e.g., Prevotella sp. MSX73) are enriched in DKD patients and contribute to a combinatorial biomarker model (AUC = 0.889) differentiating DN from T2DM without nephropathy 22. The decline in Prevotella-associated butyrate production further exacerbates renal dysfunction by impairing anti-inflammatory pathways (22, 23).

Comprehensive integration of microbial signatures (e.g., Prevotella copri, Prevotella_9, and g_Prevotella) with serum metabolites (e.g., imidazolepropionic acid) enhances early DN detection (AUC >0.94) (22, 23). This multi-omics approach underscores the potential for developing non-invasive diagnostic models leveraging GM dysbiosis (3, 24).

### 2.2 Unresolved causality: insights from MR studies

MR has become a crucial approach for establishing causality in the relationship between GM and DN. While accumulating evidence from MR analyses suggests the existence of causal links at the genetic level, several key areas remain contentious, particularly concerning the directionality of effects and the clarification of underlying molecular mechanisms.

#### 2.2.1 Evidence supporting a causal relationship

Multiple two-sample MR studies have provided substantial evidence supporting a causal relationship between specific gut microbial taxa and the risk of DN. For instance, studies have identified potential causal links between certain gut microbial taxa and DN, which were confirmed through MR analysis (25–27). Quantitative evaluations have further shown that an increased abundance of taxa such as *Catenibacterium* is significantly associated with a decreased risk of DN (OR =0.513; 95% CI, 0.343–0.768) (26, 27). Moreover, MR analyses have revealed that genetic variants influencing the composition of the gut microbiome, ranging from phylum to genus level, have both positive and negative causal effects on the risk of DN (27, 28). These data reinforce the view that gut microbial dysbiosis may act as a potential driver in DN pathogenesis. Robust causal associations have also been validated by MR studies employing inverse variance weighted methods (28).

##### 2.2.1.1 Controversy: the bidirectionality of causality

Despite the above findings, one of the major points of contention is the potential bidirectional nature of the relationship between GM dysbiosis and DN. On the one hand, dysbiosis may be an initiating factor in DN: reduced production of SCFAs has been shown to promote renal inflammation and fibrosis, thereby accelerating DN progression (9, 12). Experimental studies have demonstrated that SCFA supplementation can attenuate renal fibrosis in DN models, supporting the causal role of the GM in DN onset (5). On the other hand, renal dysfunction itself may influence the gut microbiome: studies have found that declining renal function, such as a decreased glomerular filtration rate, can alter microbial diversity (29, 30). As such, reverse causation becomes a possibility, making it challenging to fully distinguish causality using MR analysis alone (31). To address this issue, Li Q has emphasized the need for longitudinal studies, such as repeated measures of renal function, to clarify the temporal and causal relationships (3). Additionally, fecal microbiota transplantation (FMT) experiments provide direct evidence for this interaction: transplantation of microbiota from patients with DKD increased the urinary albumin-to-creatinine ratio (UACR) in recipient animals by approximately 2.1-fold compared with transplantation from healthy donors, further supporting the role of dysbiosis as a driving factor in DN (32).

##### 2.2.1.2 Controversy: unclear molecular mechanisms

Another unresolved issue lies in the lack of detailed understanding of the molecular mechanisms underlying GM-host interactions in DN. Although numerous studies have postulated that GM-derived metabolites, such as SCFAs, may modulate host pathways—including the renin-angiotensin system (RAS)—direct experimental evidence remains limited. For example, Huang L speculated that SCFAs might influence DN progression via immunometabolic pathways, but this has yet to be verified. Similarly, while Das S investigated the protective effects of SCFAs in DN models, their mechanisms—such as possible regulation of the RAS—require further elucidation (5, 12). As noted by Jin Y, “the precise pathways by which dysbiotic microbiota may induce and exacerbate DN remain undefined,” underscoring the need for mechanistic studies in cellular and animal models to move beyond mere genetic associations (27). Although FMT studies revealing elevated UACR in recipient animals offer a useful starting point for exploring these mechanistic links, the downstream pathways—potentially involving inflammation or metabolic disturbances—have not yet been clearly defined.

### 2.3 Cross-disease comparison: microbial differences between DN and NDKD

Comparative analysis between DN and NDKD reveals disease-specific patterns of GM dysbiosis, which are critical for understanding pathogenesis and guiding personalized treatment strategies. Current evidence indicates that DN and NDKD display distinct alterations in both metabolic and pathogenic microbial taxa, resulting in different responses to microbial interventions.

#### 2.3.1 Disease-specific microbial features in DN

##### 2.3.1.1 Metabolic dysfunction

DN is characterized by a notably greater depletion of short-chain fatty acid (SCFA)-producing bacteria, such as butyrate-producing Firmicutes species, compared to NDKD. Experimental studies have shown that DN mouse models exhibit significantly lower fecal SCFA levels (especially acetic acid), which correlate positively with the severity of renal fibrosis (6). Furthermore,it has been summarized that this reduction is strongly associated with insulin resistance—a relationship more pronounced in DN than in NDKD—and may impact glucose homeostasis through the “gut-kidney axis” (17). The observational data further confirm that gut microbial metabolic disturbances are a fundamental and unique aspect of DN, with SCFA depletion representing a core feature rather than a common trait of NDKD (25).

##### 2.3.1.2 Differences in pathogenic bacteria

The enrichment of pathogenic bacteria also differs between DN and NDKD. In NDKD, dysbiosis mainly affects immune-modulating bacteria; for instance, enrichment of *Clostridium* has been shown to activate immune pathways and contribute to nephritis (1). In contrast, DN is characterized by an increase in metabolically relevant pathogenic taxa, notably *Enterobacteriaceae* (6, 25). Specifically, studies have identified immune dysregulation-associated dysbiosis in NDKD (e.g., Clostridium perfringens *Clostridium* enrichment disturbing T cell balance), whereas in DN, the dysbiosis is more strongly related to metabolic pathogens such as *Enterobacteriaceae*, which can directly aggravate insulin resistance and renal injury (1). Systematic review corroborates these findings, indicating that DN features more pronounced shifts in metabolic taxa, while NDKD primarily exhibits changes in immune-related microbes (33).

Therapeutic Implications: Microbiota-targeted interventions appear to be more effective in DN, underscoring the value of disease-specific strategies. For example, butyrate supplementation significantly alleviated renal fibrosis in DN models (6), and SCFA treatment was found to slow DN progression by correcting metabolic imbalances (34). However, similar interventions in NDKD yielded limited results; studies (17, 31) point out that the pathogenesis of NDKD is primarily immune-mediated, making SCFA supplementation less effective in mitigating immune-driven kidney damage. These findings emphasize the importance of tailoring microbial interventions to the underlying pathophysiology of each kidney disease subtype.

### 2.4 Key controversies and knowledge gaps

Despite significant advances in understanding the gut microbiota - diabetic nephropathy relationship, several critical controversies and knowledge gaps persist. First, while MR studies provide genetic evidence of causality, experimental validation using animal models remains essential. Integrated studies have demonstrated associations between clinical GM profiles and DN, yet *in vivo* models are necessary to elucidate causal mechanisms (33). Although FMT represents a promising therapeutic approach, standardized protocols for DN are lacking, necessitating additional animal studies to validate causal relationships through approaches such as transplanting DN-associated microbiota into healthy recipients (17, 35).

The translation of microbiota-targeted therapies to clinical practice faces significant challenges. Clinical trials of probiotics and prebiotics have yielded inconsistent results, likely reflecting inter-individual microbiota variability and disease heterogeneity. While short-chain fatty acid (SCFA) supplementation demonstrates benefits in DN animal models (34), FMT outcomes in human studies remain variable due to substantial individual microbiome diversity (35). Recent investigations have proposed personalized probiotic formulations based on microbiome profiling; however, population variability and the need for standardized interventions continue to impede clinical translation (35, 36).

Furthermore, emerging evidence suggests that DN and NDKD involve distinct dysbiosis-driven immunological mechanisms. DN-associated gut dysbiosis appears to influence host immunity through metabolic-immune crosstalk, whereas NDKD may be more directly linked to immune dysregulation, particularly Th17/Treg cell axis imbalances (1). Although experimental studies demonstrate that microbiota modulation can restore Treg/Th17 balance in inflammatory conditions, these models lack kidney disease specificity. Consequently, comparative mechanistic studies examining these immunological pathways and their roles in renal fibrosis across DN and NDKD represent critical research priorities requiring further investigation.

## 3 Key Mechanisms by which GM dysbiosis drives the progression of DN

### 3.1 Disruption of the intestinal barrier and translocation of endotoxins

#### 3.1.1 Gut microbiota dysbiosis and intestinal barrier impairment

In the context of diabetes, GM dysbiosis is typified by a notable reduction in beneficial bacteria—particularly those producing SCFAs—accompanied by an overgrowth of Gram-negative pathogenic taxa (9, 12). This altered microbial composition leads to the downregulation of critical tight junction proteins within intestinal epithelial cells, such as ZO-1, occludin, and claudin-1, thereby compromising the integrity of the intestinal mucosal barrier (37–39) and resulting in enhanced intestinal permeability (40). Compelling evidence has demonstrated a depleted abundance of protective genera, such as *Akkermansia*, and an increased proportion of LPS-producing Gram-negative bacteria (Such as Bacteroides stercoris) in the gut of patients with DN (23). Animal studies further corroborate that high-fat diet or diabetic status markedly reduces the expression of tight junction proteins and exacerbates gut barrier dysfunction (41, 42).

#### 3.3.2 Endotoxemia and activation of systemic inflammatory responses

Intestinal barrier disruption allows bacterial endotoxins, particularly LPS, to enter the systemic circulation, leading to metabolic endotoxemia (40, 43). Circulating LPS engages Toll-like receptor 4 (TLR4) on monocytes and macrophages, triggering the MyD88/NF-κB signaling pathway (25, 44, 45). This activation induces the release of pro-inflammatory cytokines, including TNF-α and IL-6 (25, 46). Clinical investigations have demonstrated a positive correlation between serum LPS levels and the degree of renal injury in DN, including declines in glomerular filtration rate and aggravation of proteinuria (3).

#### 3.3.3 Renal inflammation and fibrosis

**Figure 1:**
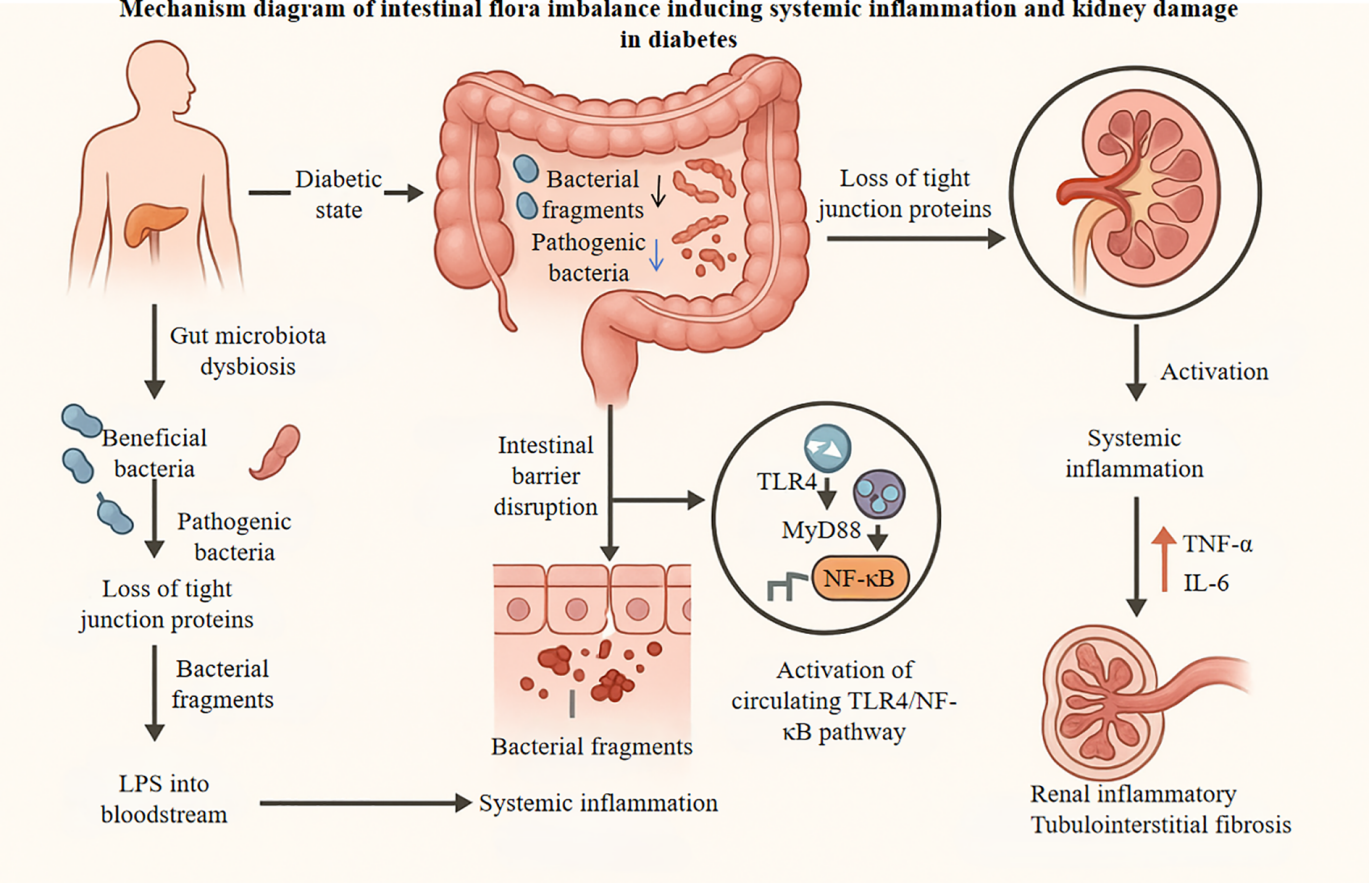
Mechanism of gut microbiota dysbiosis-induced systemic inflammation and renal damage in diabetes. Left: Diabetic conditions drive gut microbiota dysbiosis, characterized by diminished beneficial bacteria (blue) and expansion of pathogenic bacteria (red). This imbalance disrupts intestinal tight junctions, permitting translocation of bacterial fragments (e.g., LPS) into systemic circulation. Center: Key molecular pathway: Circulating bacterial fragments activate the TLR4/MyD88/NF-kB signaling cascade (detailed in dashed- line inset), triggering release of pro- inflammatory cytokines (TNF-α, IL-6). Right: Systemic inflammation propagates renal injury, culminating in inflammatory tubulointerstitial fibrosis. TLR4, Toll-like receptor 4; MyD88, Myeloid differentiation primary response 88; NF-κB, Nuclear factor kappa-light- chain-enhancer of activated B cells; TNF-a, Tumor necrosis factor-alpha; IL-6, Interleukin-6; LPS, Lipopolysaccharide.

Systemic inflammatory cytokines, including TNF-α and IL-6, drive the infiltration of immune cells into renal tissue and activate local inflammatory pathways (17). Inflammatory responses cause direct damage to glomerular endothelial cells and podocytes, accelerate the progression of glomerulosclerosis, and promote the transdifferentiation of tubular epithelial cells into interstitial fibroblasts, thereby exacerbating tubulointerstitial fibrosis (1, 18). Experimental evidence suggests that interventions using probiotics or plant-derived extracts can effectively mitigate renal inflammation and fibrosis in diabetic models. Specifically, probiotic supplementation, such as Lactobacillus ATCC 4356, has been shown to reduce kidney inflammation and fibrosis in diabetic rats by modulating GM, restoring microbial diversity, and decreasing markers of DNA damage, as evidenced by histological analyses that revealed improved kidney structure and reduced fibrosis (47). Additionally, probiotic combinations including strains like Lactobacillus TYCA06 and Bifidobacterium BLI-02 attenuate renal function deterioration and blood-glucose fluctuations in diabetic CKD models, thereby suppressing inflammatory pathways (48). For plant-derived extracts, bioactive compounds from medicinal plants, such as flavonoid-rich preparations, demonstrate nephroprotective effects by suppressing oxidative stress and regulating proinflammatory pathways (e.g., through modulation of sirtuins and claudin-1 expression), thus reducing renal injury in streptozotocin-induced DN (48, 49). These interventions work through mechanisms like inhibition of inflammatory signaling cascades and modulation of the gut-kidney axis, as they reduce pro-inflammatory cytokines and oxidative stress markers, contributing to the amelioration of fibrosis (36, 50). The specific mechanistic process is summarized in **Figure 1**.

### 3.2 Nephrotoxic effects of microbial metabolites

Gut-derived microbial metabolites, particularly the reduction of SCFAs and the accumulation of uremic toxins, play critical roles in the development and progression of DN. Their pathogenic mechanisms involve oxidative stress, inflammatory responses, and fibrosis, in part through modulation of key renal signaling pathways (44, 51).

#### 3.2.1 Pathogenic mechanisms of reduced SCFAs

A decrease in SCFAs, such as butyrate and propionate, exacerbates renal injury primarily by impairing renal antioxidant defense systems and disrupting immune homeostasis. SCFA deficiency attenuates the activation of major regulatory pathways, such as nuclear factor erythroid 2-related factor 2 (Nrf2), leading to the accumulation of reactive oxygen species (ROS) and enhancing oxidative stress within the renal microenvironment (52). Reduced SCFA levels impair immunomodulatory functions by inhibiting regulatory T cell (Treg) differentiation and decreasing anti-inflammatory cytokines such as IL-10. This shift promotes the production of pro-inflammatory mediators, including IL-1β, IL-6, and TNF-α, leading to sustained renal inflammation and tissue damage (6, 44, 53). This immune dysregulation is increasingly recognized as a pivotal feature in the interplay between GM and renal pathology (51, 54).

#### 3.2.2 Nephrotoxic mechanisms of uremic toxin accumulation

Uremic toxins, such as indoxyl sulfate (IS) and p-cresyl sulfate (PCS), exhibit a strong inverse correlation with estimated glomerular filtration rate (eGFR) (total IS: r = -0.819; free PCS: r = -0.753) (55), further aggravate renal damage in DN by promoting mitochondrial dysfunction and profibrotic remodeling. IS activates the aryl hydrocarbon receptor (AhR) pathway in renal tubular epithelial cells, enhancing oxidative stress, impairing mitochondrial function, and inducing apoptosis (53, 54, 56).Critically, serum IS ≥50 μmol/L predicts peritoneal dialysis technique failure with 70.4% sensitivity and 87.9% specificity (p<0.0001), highlighting its clinical relevance in renal functional decline 57. PCS, on the other hand, drives fibroblast activation and extracellular matrix deposition, thereby accelerating tubulointerstitial fibrosis (54, 57). Moreover, IS accumulation downregulates organic anion transporter 3 (OAT3) expression at the blood-brain barrier, impairing toxin efflux and exacerbating systemic accumulation (58). Importantly, IS and PCS can synergistically activate the NF-κB signaling pathway, upregulating pro-inflammatory cytokines such as IL-17 and IL-6, which directly correlate with peritoneal dialysate IL-6 levels (r = 0.92) (59), perpetuating a vicious cycle of inflammation and fibrosis (51, 57).

In DKD, GM dysbiosis promotes uremic toxin production (e.g., IS/PCS) (60, 61), while AST-120 adsorbent therapy significantly lowers serum IS (SMD = -1.75, p<0.001) and improves creatinine clearance (SMD = 0.41, p<0.001) in CKD models (62). This dysbiosis compromises intestinal barrier function, facilitating systemic toxin entry and exacerbating renal inflammation8,16. Dietary modulation (e.g., high-fiber diets, resistant starch) reduces toxin generation by altering microbiota composition (60, 63, 64), effectively lowering serum total cholesterol (SMD = -0.28, p=0.013) in diabetic CKD models (62), thereby mitigating DKD progression.

#### 3.2.3 Interventional strategies and therapeutic potential

Targeted interventions aimed at restoring microbial metabolic balance have shown promising renoprotective effects, mainly through pharmacological, nutritional, and non-pharmacological approaches.

##### 3.2.3.1 Pharmacological and nutritional modulation

Traditional Chinese herbal formulations (e.g., Xiaoyu Xiezhuo decoction, XXD) have been reported to lower plasma IS and PCS levels while increasing colonic SCFA (such as butyrate) concentrations, consequently ameliorating tubular injury and suppressing pro-inflammatory cytokines such as IL-17 and TNF-α (65, 66). Specific probiotic strains (e.g., ATCC 4356) facilitate the re-establishment of microbial diversity and enhance SCFA biosynthesis, thus mitigating oxidative stress and glomerulosclerosis (47). Similarly, natural compounds like resveratrol may reshape the GM structure, elevate fecal SCFA levels, and reduce tubulointerstitial fibrosis (6).

##### 3.2.3.2 Non-pharmacological toxin removal

Use of intestinal adsorbents such as AST-120 effectively reduces circulating IS by binding its precursors in the gut, while extracorporeal techniques including hemodialysis and hemofiltration directly eliminate accumulated IS and PCS from the bloodstream (54). These interventions, by targeting the gut–kidney axis, present innovative strategies for metabolic regulation and renoprotection among patients with DN (44, 57).

### 3.3 Activation of immune-inflammatory pathways

GM dysbiosis triggers a cascade of immune-inflammatory responses through both innate and adaptive mechanisms, with the NLRP3–IL-1β axis serving as a central regulatory hub. Dysbiosis increases host exposure to endotoxins such as LPS and uremic toxins, which enter systemic circulation and function as pathogen-associated or damage-associated molecular patterns (42, 67–69). These molecules initiate innate immune activation through a two-signal process: LPS activates the TLR4/NF-κB pathway (Signal 1), inducing transcription of NLRP3 and pro-IL-1β, while uremic toxins trigger potassium efflux and mitochondrial ROS production (Signal 2), promoting NLRP3 oligomerization and caspase-1 activation (69–71).

This activation specifically promotes assembly of the NLRP3–ASC–pro-caspase-1 complex via the P2X7 receptor, leading to caspase-1-dependent maturation of IL-1β and IL-18 (68, 69). Activated caspase-1 cleaves gasdermin D, forming membrane pores that induce pyroptosis in glomerular endothelial cells—a form of inflammatory cell death characterized by cellular swelling, lysis, and massive cytokine release, resulting in direct renal tissue injury (42, 72, 73). The ensuing inflammatory milieu recruits neutrophils into the renal parenchyma, amplifying oxidative stress and contributing to podocyte injury (42, 67, 74).

Concurrently, altered GM disrupts adaptive immune homeostasis. Reduced beneficial metabolites, particularly SCFAs, skews T cell differentiation toward excessive Th17 activation while impairing regulatory T cell (Treg) function (74, 75). Hyperactivated Th17 cells release elevated IL-17A, which acts on kidney-resident cells to induce neutrophil chemoattractants (CXCL1, CXCL2) (73, 74). Infiltrating neutrophils release proteases and ROS that degrade podocyte cytoskeleton proteins and disrupt the slit diaphragm, culminating in proteinuria (73, 74). Critically, IL-17A upregulates NLRP3 expression in glomerular cells, establishing an IL-17A–NLRP3–IL-1β positive feedback loop that perpetuates renal inflammation (73–75).

**Figure 2:**
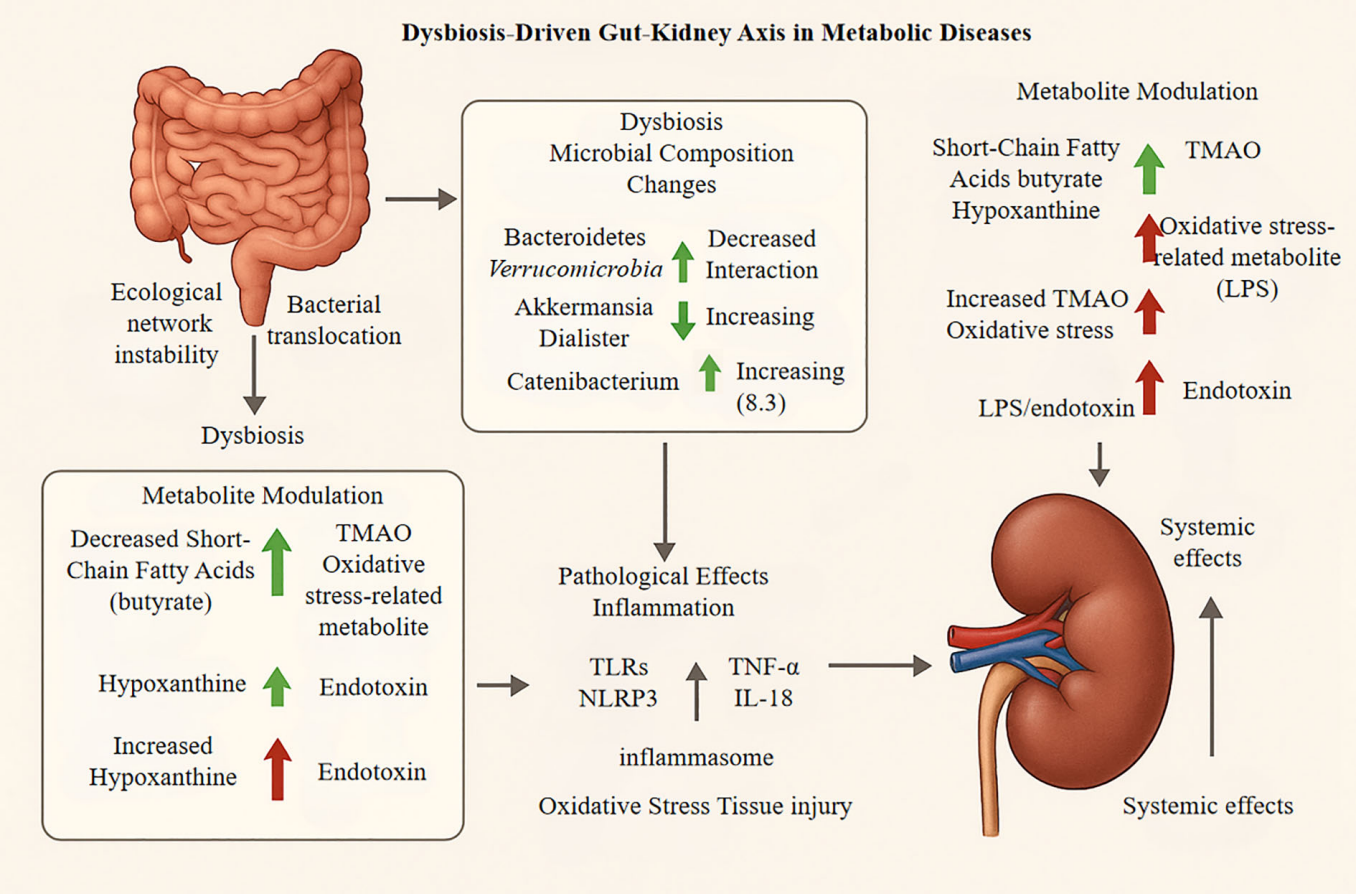
Downstream pathological effects, encompassing both renal injury and systemic consequences, involve several key processes. First, an amplification of inflammation occurs as microbial metabolites, such as lipopolysaccharide (LPS) and trimethylamine N-oxide (TMAO), activate renal-resident immune cells like macrophages. This activation triggers the release of pro-inflammatory cytokines, including TNF-α and IL-1β. Concurrently, the NLRP3 inflammasome is activated, promoting renal cell apoptosis and fibrosis. Metabolic imbalances also induce oxidative stress through the accumulation of reactive oxygen species (ROS), which damages the glomerular filtration barrier and leads to tubular epithelial cell injury and proteinuria under chronic conditions. These effects create a systemic feedback loop; for instance, a decline in the glomerular filtration rate (GFR) exacerbates toxin accumulation, which in turn worsens gut dysbiosis. This highlights complex inter- organ crosstalk, such as the kidney-brain axis, driving disease progression.

The NLRP3–IL-1β axis thus bridges innate and adaptive immunity in DN pathogenesis. Therapeutically, targeting the NLRP3/caspase-1 pathway significantly reduces cytokine release and ameliorates glomerulosclerosis and proteinuria, emerging as a core target for mitigating GM-driven inflammation in DN (67, 76). The “gut–kidney axis”—encompassing primary microbiota alterations, secondary metabolite regulation, and tertiary pathological effects—is illustrated in **Figure 2**.

### 3.4 Role of metabolic dysregulation as a mediator

GM dysbiosis drives DN progression through interconnected metabolic pathways involving insulin resistance and lipid dysregulation. The disruption of gut microbiota significantly reduces SCFA synthesis, particularly acetate and butyrate (35, 51). SCFAs normally engage G protein-coupled receptors (GPR41/43) on intestinal L cells to promote glucagon-like peptide-1 (GLP-1) secretion, which is crucial for glycemic control (34). GLP-1 deficiency diminishes its inhibitory effect on hepatic gluconeogenesis, increasing activity of rate-limiting enzymes like phosphoenolpyruvate carboxykinase (PEPCK) and glucose-6-phosphatase (G6Pase), thereby exacerbating hyperglycemia (34, 77). Notably, when serum butyrate levels fall below 10 μM, renal IL-6 expression significantly increases (34).

Concurrently, gut dysbiosis facilitates LPS translocation into systemic circulation, activating TLR4 signaling pathways (78). This triggers the JNK signaling cascade in macrophages, leading to increased tumor necrosis factor-alpha (TNF-α) release. TNF-α promotes serine phosphorylation of insulin receptor substrate-1 (IRS-1), disrupting the downstream PI3K-AKT pathway and impairing glucose uptake in peripheral tissues (7, 44, 78). The resulting chronic insulin resistance induces sustained hyperinsulinemia, which causes afferent arteriolar dilation and efferent arteriolar constriction, increasing glomerular capillary pressure. These hemodynamic alterations promote proteinuria and glomerulosclerosis, accelerating renal injury progression (11).

Parallel to glucose metabolism disruption, gut dysbiosis profoundly alters lipid homeostasis through impaired bile acid metabolism. The reduced formation of secondary bile acids, particularly deoxycholic acid, impairs farnesoid X receptor (FXR) activation in the intestine (77, 79, 80). Inadequate FXR signaling upregulates hepatic sterol regulatory element-binding protein 1c (SREBP-1c), driving transcription of lipogenic enzymes including fatty acid synthase (FAS) and acetyl-CoA carboxylase (ACC). This promotes triglyceride synthesis and hepatic lipid accumulation (77, 79, 81). Under physiological conditions, intestinal FXR activation stimulates fibroblast growth factor 19 (FGF19) secretion, which inhibits hepatic CYP7A1-mediated bile acid synthesis and preserves lipid homeostasis (35, 77). In DN, suppressed FXR signaling decreases FGF19 levels, leading to insufficient SREBP-1c inhibition and systemic lipid overload (77, 79, 81).The elevated circulating free fatty acids (FFA) resulting from this metabolic disruption enhance renal lipid deposition, triggering mesangial cell lipotoxicity and mitochondrial oxidative stress. This stimulates release of inflammatory mediators including IL-6 and monocyte chemoattractant protein-1 (MCP-1), culminating in mesangial matrix expansion (19, 81). The synergistic effects of insulin resistance and lipid dysregulation accelerate renal deterioration through multiple mechanisms: insulin resistance-induced intraglomerular hypertension combines with lipotoxicity-mediated mesangial cell injury to promote glomerular basement membrane thickening and excessive extracellular matrix accumulation (11, 19). Furthermore, concomitant activation of the JNK pathway (linked to insulin resistance) and FFA-driven NLRP3 inflammasome assembly (associated with lipid dysregulation) synergistically promotes macrophage infiltration in renal tissue, hastening tubulointerstitial fibrosis (78). This integrated metabolic dysfunction underscores the critical role of the gut-kidney axis in DN pathogenesis.

## 4 Strategies targeting the GM in the management of DN

### 4.1 Probiotics, prebiotics, and synbiotics and postbiotics

Probiotics, prebiotics, and synbiotics have gained increasing attention as microbiota-targeted interventions for the management of DN. An accumulating body of clinical evidence suggests that these approaches, through modulation of the gut–kidney axis, can reshape intestinal microbial composition, reduce oxidative stress, and ultimately improve renal function parameters such as serum creatinine and blood urea nitrogen (47, 82). Probiotics, by restoring the balance of the GM, have been shown to lower the risk of renal injury. Synbiotics, which combine the complementary actions of probiotics and prebiotics, produce synergistic effects by regulating the production of key microbial metabolites, such as SCFAs and secondary bile acids, thereby mitigating systemic inflammatory responses (35, 83, 84).

Several randomized controlled trials have demonstrated that these interventions may also promote glycemic control (evidenced by reduced HbA1c levels) and improve lipid metabolism (e.g., lowering low-density lipoprotein cholesterol), underscoring the close interplay between the GM and host metabolic pathways (84, 85). Nevertheless, substantial heterogeneity and limitations persist across studies. The therapeutic efficacy of specific bacterial strains varies, potentially due to inter-individual differences in baseline gut microbial communities (47, 82). Furthermore, clinical protocols regarding optimal dosing, strain selection, and duration of interventions are not yet standardized, thereby affecting the reproducibility and robustness of clinical outcomes.

Given these challenges, future research should prioritize large-scale, multicenter randomized controlled trials to comprehensively assess the impact of targeted microbiome interventions on hard endpoints in DN and to facilitate the development of next-generation precision microbiome therapeutics (86). In summary, while microbiota-targeted interventions represent a promising avenue for DN management, further rigorous clinical validation is warranted to substantiate their efficacy.

### 4.2 Mechanistic insights into the role of high-fiber diets in DN

Emerging evidence highlights the therapeutic role of high-fiber diets in delaying the progression of DN, primarily through modulation of GM-derived metabolites such as SCFAs and restoration of intestinal barrier integrity. The underlying mechanisms are summarized as follows:

#### 4.2.1 Enhancement of SCFA production

High-fiber diets, particularly those rich in fermentable fibers like pectin and inulin, are metabolized by colonic microbiota, leading to increased production of SCFAs such as acetate, propionate, and butyrate (53, 87, 88). These dietary interventions selectively enrich SCFA-producing bacteria, including *Akkermansia muciniphila* and members of the *Bacteroides* genus, thereby optimizing the gut microbial landscape (89, 90). Notably, butyrate has been demonstrated to improve insulin sensitivity and alleviate renal fibrosis and inflammatory responses by inhibiting HDACs (14, 34).

#### 4.2.2 Restoration of intestinal barrier function

SCFAs are crucial for intestinal barrier integrity by enhancing tight junction protein expression, which decreases endotoxin translocation, such as LPS, into systemic circulation (10, 34, 91). They activate GPR41/43, aid regulatory T cell (Treg) differentiation, and increase anti-inflammatory cytokines like interleukin-10 (IL-10), thereby reducing systemic inflammation (92–94). In preclinical models, these effects are associated with marked decreases in proteinuria and tubular injury (14, 34, 95).

#### 4.2.3 Modulation of the gut–kidney axis

High-fiber dietary interventions have been shown to rebalance GM composition, characterized by a reduction in pathogenic bacteria such as *Enterobacter* and an increase in beneficial taxa like *Bacteroidetes* (1, 3, 5). Through the action of SCFAs, these interventions further inhibit renal RAS activation and attenuate oxidative stress in renal tissues (14, 96). Clinical studies corroborate these findings, demonstrating that high-fiber interventions can significantly decrease glycemic parameters (e.g., reduction in HbA1c by 0.99%) and inflammatory markers in patients with DN (97). Nevertheless, individual variability in gut microbiome composition warrants personalized optimization of fiber type, with current evidence suggesting that soluble fibers confer greater efficacy (98).

In summary, high-fiber diets exert multifaceted renoprotective effects in DN via the microbiota–SCFA–intestinal barrier axis, ultimately ameliorating pathological progression. Future directions should focus on the development of individualized fiber-based dietary strategies and targeted SCFA delivery approaches to maximize therapeutic benefit.

### 4.3 Regulatory effects of microbiota-derived metabolites: the renoprotective role of butyrate

Butyrate, a key short-chain fatty acid derived from gut microbial fermentation, has shown notable renoprotective effects in multiple animal models of DKD and related renal injuries (34, 99). Studies have demonstrated that butyrate supplementation can markedly alleviate glomerular hypertrophy, podocyte injury, and interstitial fibrosis, as well as improve mitochondrial function and reduce serum creatinine levels in both diabetic and acute kidney injury models (100, 101). These results suggest that targeting microbial metabolites like butyrate may offer novel strategies for preserving kidney function.

Mechanistic investigations indicate that butyrate exerts its renoprotective roles via several pathways. It effectively suppresses renal inflammation and oxidative stress by downregulating pro-inflammatory mediators, such as TNF-α, and inhibits fibrosis by modulating TGF-β signaling in renal tubular cells and podocytes through GPR43 and GPR109A receptors (34, 102). In addition, butyrate acts as a histone deacetylase inhibitor, promoting beneficial epigenetic modifications and upregulating protective genes including Klotho and PGC-1α (100, 103), thereby restoring mitochondrial homeostasis primarily via the AMPK/PGC-1α pathway (80, 104).

Furthermore, butyrate reinforces the gut–kidney axis by enhancing intestinal barrier integrity and attenuating systemic inflammation, with evidence pointing to a receptor-dependent mechanism and the need for sustained supplementation to maintain its benefits (105, 106). In an adenine-induced model of CKD, butyrate has been shown to attenuate renal fibrosis by suppressing activation of the NLRP3/STING/NF-κB signaling pathway (107, 108). Collectively, these findings highlight the therapeutic potential of butyrate in DKD, supporting further clinical studies to optimize its application in patient care.

### 4.4 Exploration of novel targeted therapies

In recent years, increasing attention has been paid to the interaction between DKD and the GM, leading to the exploration of novel targeted therapeutic strategies. These strategies, primarily based on natural compounds and traditional Chinese medicine interventions, aim to regulate gut microbial homeostasis through multi-target mechanisms. Such interventions not only help restore microbial balance but also alleviate systemic inflammation and metabolic disturbances, ultimately contributing to the delay of DKD progression (109, 110).

#### 4.4.1 Intervention with natural compounds

##### 4.4.1.1 Intervention with natural compounds

Natural compounds demonstrate significant potential in modulating the gut microbiome and ameliorating DKD progression, primarily by regulating microbial-derived metabolites. Clinical and preclinical studies consistently report decreased abundance of probiotic genera Bifidobacterium and Lactobacillus (e.g., L. johnsonii, L. reuteri, B. animalis) in patients with CKD including DKD, which correlates with impaired renal function and increased uremic toxins (111, 112). For instance, plant polysaccharides and related natural products reshape GM composition, enriching SCFA-producing bacteria such as Bifidobacterium and Lactobacillus (35, 113, 114). TCM interventions like Moshen granule and specific compounds (e.g., barleriside A, 5,6,7,8,3’,4’-hexamethoxyflavone, Thonningianin A) counteract this dysbiosis by selectively increasing Lactobacillus/Bifidobacterium abundance, restoring microbial balance and gut barrier integrity (115, 116). This modulation reduces accumulation of diverse uremic toxins implicated in DKD pathogenesis, including:

Phenylacetylglutamine (PAGln): Derived from phenylalanine metabolism by gut microbes (e.g., Clostridium spp.), PAGln promotes cardiovascular complications and renal fibrosis via activation of G-protein-coupled receptors, exacerbating DKD progression (117).

p-Cresyl glucuronide and PCS: Protein-bound toxins produced from tyrosine metabolism by Bacteroides and Clostridium. They induce oxidative stress, endothelial dysfunction, and insulin resistance, accelerating renal injury (118, 119).

Hippuric acid: Generated from polyphenol metabolism (e.g., quercetin, chlorogenic acid), it contributes to tubular damage and inflammation in diabetic kidneys (119).

These interventions further suppress proteobacteria (e.g., Escherichia-Shigella) and reduce LPS release, thereby mitigating systemic inflammation via NLRP3/ASC/Caspase-1 pathway inhibition (116). TMAO, a gut-derived metabolite, remains a key contributor to renal injury in DKD. Elevated TMAO levels correlate strongly with glomerular filtration rate decline (117, 119). Natural polyphenols (e.g., resveratrol, curcumin) suppress TMAO generation by inhibiting microbial trimethylamine (TMA) production and hepatic flavin monooxygenase activity, thereby mitigating renal oxidative stress and inflammation (17, 114).

Collectively, TCM-based modulation of the gut-kidney axis operates through a “microbiota-metabolite-inflammation” cascade (1): Correction of dysbiosis enriches beneficial taxa (2); Restoration of gut barrier reduces toxin translocation (3); Downregulation of inflammatory pathways (e.g., NLRP3, AhR) alleviates renal fibrosis (120, 121). The multitargeted actions of natural compounds constitute their primary advantage: they concurrently enhance microbial diversity (e.g., reducing Enterobacteriaceae while increasing SCFA producers), directly scavenge uremic toxins, and modulate host immune pathways (e.g., NLRP3 inflammasome suppression). These findings underscore microbiota–kidney axis-targeted interventions using natural compounds as a promising strategy for DKD management (3, 114, 118).

#### 4.4.2 Potential of traditional Chinese medicine interventions

TCM has demonstrated distinct advantages in the regulation of GM for the management of DN. Mounting evidence highlights that TCM interventions target intestinal-derived metabolites (e.g., SCFAs, tryptophan derivatives, and LPS) through microbiota remodeling, thereby ameliorating gut-kidney axis dysregulation (116, 122). Owing to its multi-component and multi-target characteristics, TCM can simultaneously enhance intestinal barrier integrity, suppress the release of inflammatory mediators, and attenuate renal injury. Accumulating evidence from clinical and preclinical studies suggests that the therapeutic effects of TCM compound prescriptions are primarily mediated by modulation of gut microbial composition and SCFA metabolism.

Recent studies elucidate that TCM formulas significantly modulate tryptophan metabolism pathways. For instance, *Tang Shen Formula* (TSF) reduces accumulation of uremic toxins (IS and p-CS) by enriching *Lactobacillus* and *Bifidobacterium*, while increasing serum indole-3-aldehyde (IAld)—an AHR ligand that inhibits renal inflammation and fibrosis (1, 3). Similarly, *Shenyan Kangfu Tablet* downregulates gut-derived TMAO and suppresses NLRP3 inflammasome activation via the LPS-TLR4 pathway (55). For example, Shenqi Dihuang Decoction has been shown to increase the abundance of *Roseburia* in the GM, thereby promoting the production of SCFAs such as butyrate. This modulation improves intestinal barrier function, reduces pro-inflammatory cytokines such as TNF-α and IL-6, and effectively alleviates renal fibrosis (123, 124). Notably, natural compounds like *Thonningianin A* (from *Penthorum chinense*) ameliorate intestinal barrier impairment by restoring tight junction proteins (claudin-1, occludin, ZO-1), subsequently reducing fecal and serum LPS levels and inhibiting renal NLRP3/ASC/caspase-1 signaling (125). In clinical practice, Huangkui Capsule has demonstrated efficacy in lowering serum creatinine and blood urea nitrogen levels. The underlying mechanism is closely related to the inhibition of gut-derived uremic toxin generation, as well as the suppression of inflammation, consequently mitigating renal damage (110, 126). A multicenter trial further confirms that *Yi-Shen-Hua-Shi Granule* reduces proteinuria by elevating anti-inflammatory indole-3-propionic acid (IPA) and decreasing pro-fibrotic kynurenine, highlighting the role of tryptophan metabolism in DN progression (116). Qiditangshen Granules, by targeting the GM–SCFA axis and activating receptors such as GPR43, can inhibit overactivation of the renin-angiotensin system (RAS), thereby reducing glomerulosclerosis and tubular injury (123, 126).

The holistic approach of TCM thus addresses the multifaceted pathophysiology of DN through coordinated regulation of gut microecology, microbial metabolite profiles (SCFAs, tryptophan derivatives, LPS), and immune homeostasis (123, 124, 126). Such interventions not only restore microbial diversity but also attenuate inflammation and oxidative stress by modulating metabolite-mediated pathways (e.g., AHR/NF-κB, NLRP3, RAS) (55, 116, 122, 125). Future research should aim to further elucidate the specific molecular mechanisms through which TCM regulates GM, with the goal of optimizing clinical interventions. (For details of relevant clinical TCM studies, see **Table 1**).

**Table 1:** Summary of literature on the modulation of gut microbiota by natural compounds and Traditional Chinese Medicine (TCM).

Corresponding author	Year	Study type	Sample size	Intervention type	Core mechanism	Key evidence source
Pan LM	2024	Review	Not Stated	Traditional Chinese Medicine (TCM)	Modulation of gut microbiota	Literature review and summary
Wang Y	2024	Review	Not Stated	No intervention	Gut microbiota dysbiosis, altered metabolites, immune-inflammation	Literature review and mechanistic discussion
Li X	2024	Review	Not Stated	TCM	Modulation of gut microbiota, metabolites, and related signaling pathways	Literature review and summary
Tian Z	2023	Bibliometric Analysis	1009 publications	No intervention	No intervention	VOSviewer analysis of literature data
Dong Y	2025	Bibliometric Analysis	1289 publications	No intervention	No intervention	CiteSpace & VOSviewer analysis of literature data
Zhang G	2024	Review with Evidence Mapping	139 studies	TCM	No intervention	Summary and analysis of clinical evidence
Gong YX	2025	Review	Not Stated	Active components of TCM	Inhibition of renal tubular epithelial cell apoptosis	Literature review and summary
Feng Z	2025	Bibliometric Analysis	1585 publications	TCM-mediated gut microbiota regulation	No intervention	CiteSpace & VOSviewer analysis of literature data
Du J	2024	Bibliometric Analysis	711 publications	TCM	No intervention	VOSviewer & CiteSpace analysis of literature data
Wang Y	2024	Review	Not Stated	TCM, Gut microbiota	Gut-lung axis regulation	Literature review and mechanistic discussion
Wu J	2023	Preclinical Study	Non-obese diabetic (NOD) mice	*Abelmoschus manihot*	Modulation of gut microbiota and circulating metabolites	16S rRNA sequencing, metabolomics
Chang H	2024	Review	Not Stated	Chinese herbal medicine	No intervention	Review of clinical evidence and potential mechanisms
Xue M	2025	Preclinical Study	db/db mice	Danggui Buxue decoction	Regulation of autophagy via the miR-27a/PI3K/AKT pathway	Animal model experiments
Liu Y	2022	Network Pharmacology	N/A	Yishen capsules	Multi-target, multi-pathway regulation	Analysis of network pharmacology databases and software
Xu J	2024	Review	Not Stated	Edible TCM	Modulation of gut microbiota metabolites	Literature review and summary
Xu D	2024	Review	Not Stated	TCM	Modulation of gut microbiota and the microbiota-gut-x axis	Literature review and summary
Han J	2022	Review	Not Stated	Herbal medicine	Modulation of gut microbiota	Literature review and summary
Qin Y	2024	Systematic Review & Meta-analysis	30 RCTs (2306 patients)	TCM decoction	No intervention	Meta-analysis of randomized controlled trials (RCTs)
Gao S	2024	Preclinical Study	db/db mice	Jiang Tang San Hao Formula	Affecting the gut-microbiota-brain axis	16S rRNA sequencing, metabolomics, behavioral tests
Zhang L	2024	Review	Not Stated	TCM	Adjusting gut microbiota to improve immune imbalance	Literature review and summary
Chen Y	2023	Review	Not Stated	TCM	Gut microbiota-based therapy against bacteria	Literature review and summary
Tao P	2025	Network Pharmacology	N/A	Gut microbiota metabolites	Synergistic effects of multi-component, multi-target interactions	Analysis of databases such as TCMSP and DrugBank
Yang J	2023	Review	Not Stated	Gut microbiota metabolites	No intervention	Literature review and mechanistic discussion
Hui S	2024	Preclinical Study	db/db mice	Resveratrol	Modulation of the gut microbiota-short-chain fatty acids (SCFAs) axis	16S rRNA sequencing, targeted metabolomics

### 4.5 Potential value of FMT in DN

FMT has demonstrated significant therapeutic potential in the management of DN by facilitating the reconstruction of a healthy gut microbial ecosystem and modulating relevant metabolic and immune pathways (36, 127). The current evidence is summarized below from aspects of efficacy in animal models and the progress and challenges of clinical translation.

#### 4.5.1 Efficacy in animal models

Experimental studies have provided robust evidence that FMT can improve renal outcomes and restore intestinal microbial homeostasis in DN models. For instance, in DN animal models such as 5/6 nephrectomized rats, FMT intervention has been shown to remodel gut microbial composition, ameliorate dysbiosis, and attenuate glomerulosclerosis and interstitial fibrosis by regulating metabolic pathways such as serum amino acid metabolism (128, 129). Similarly, in type 1 diabetes models, FMT improved the overall metabolic profile through modulation of the gut-metabolic axis, which indirectly reduced the risk of renal injury (130, 131). These findings underscore the role of FMT in restoring microbial diversity and suppressing DN progression, while also providing mechanistic insights for future investigations (17, 129).

#### 4.5.2 Clinical translation: advances and challenges

Preliminary clinical data suggest that FMT may delay the progression of CKD, including diabetes-related CKD, highlighting its potential as an alternative approach to restore GM balance (132, 133). However, several key obstacles remain to be overcome in clinical practice:

(1) Safety concerns: The risk of infection associated with FMT procedures and uncertainty regarding long-term safety profiles represent major challenges that require careful evaluation and ongoing surveillance (128, 134). (2) Standardization: The absence of unified protocols for the preparation and delivery of FMT, such as microbial capsules, hampers the reproducibility and consistency of therapeutic outcomes across studies (135, 136). (3) Mechanistic ambiguity: Immunoregulatory and metabolic benefits observed in preclinical models need further substantiation, as the causal pathways and long-term efficacy in humans remain unexplored. High-quality randomized controlled trials are warranted to address these gaps (18).

In summary, while FMT holds considerable promise for the treatment of DN—as substantiated by preclinical evidence—its broader application requires further optimization of safety, standardization of protocols, and mechanistic clarification. Future research combining multi-omics approaches with large-scale clinical studies will be essential to advance the development of precise FMT-based interventions for DN management.

## 5 Current limitations and challenges in research

### 5.1 Ambiguity of causal relationships

The ambiguity of causal relationships stands as a central bottleneck in current research on DN and GM, significantly impeding clinical translation. This ambiguity arises from several layers of complexity.

Firstly, most available evidence is derived from observational studies, which inherently lack the ability to establish definitive causal links. For example, cross-sectional designs have reported alterations in GM abundance among DN patients (such as fluctuations in specific microbial taxa) (26, 28). However, such methodologies are inherently limited in clarifying the directionality of causation or adequately accounting for confounding factors such as dietary patterns or pharmacotherapy (18). As highlighted in the literature, “despite emerging evidence supporting an association, the causal relationship between them hasn’t been clarified yet”, underscoring that correlational findings alone are insufficient to define the underlying mechanism of causality (18).

Secondly, the confounding effect of extraneous variables and the possibility of reverse causality further exacerbate this uncertainty. Factors such as metabolic status may generate spurious associations (137), while reverse causality—whereby DN may induce gut microbial dysbiosis—complicates the interpretation of the microbiota as either a pathogenic driver or a biomarker (18, 137). This is exemplified by reverse MR analyses: as reported in the literature (18), such analyses assessing the impact of DN on GM “did not identify any significant associations”, suggesting that observed correlations could be driven by reverse causality rather than direct causative effects.

Finally, while MR represents a promising tool for disentangling causality, practical limitations persist. MR leverages genetic variants (e.g., GM-related SNPs) to minimize confounding (18, 138); however, its utility is constrained by the strength and heterogeneity of the instrumental variables. For instance, forward MR analyses have identified putative causal effects of certain gut microbes (18, 124), yet reverse MR analyses often yield null results, highlighting ongoing difficulties in resolving the precise directionality of the relationship. In summary, such causal ambiguity remains a major limitation hindering the development of microbiota-targeted therapies in DN, emphasizing the need to integrate more robust MR approaches for advanced causal inference.

### 5.2 Fragmentation of mechanistic studies: a systemic lack of integration among metabolic, immune, and barrier pathways

Despite considerable advances in DN research, the mechanistic understanding remains fragmented, particularly regarding the integration of metabolic, immune, and barrier pathways. Current studies predominantly address these axes independently, resulting in a lack of comprehensive and unified framework for DN pathogenesis.

Research on metabolic pathways exemplifies this segregation. While GM-derived metabolites such as pyrroline and glycine-conjugated bile acids have been causally linked to DN progression (139), these investigations focus primarily on the microbiota–metabolite axis without addressing interactions with immune responses or barrier integrity. Potential synergistic mechanisms remain largely unexplored (51, 139). Similarly, immunoinflammatory activation, including NLRP3 inflammasome activation and macrophage infiltration, represents a well-established driver of DN (140, 141). However, most studies assess immune factors in isolation, seldom considering how immune cell dynamics interact with intestinal or renal barrier disturbances or fluctuations in microbial metabolites (139, 142). Although gut-kidney axis activation has been shown to modulate immunity (143), comprehensive elucidation of how metabolites modulate immune signaling through TLR pathways leading to barrier dysfunction remains lacking (139, 141). The investigation of barrier integrity follows a similarly narrow approach. Impairment of tight junction proteins and resulting dysfunctions of intestinal and glomerular barriers are hallmarks of DN (144, 145). Yet current research often fails to dissect how barrier injury is co-regulated by both microbial metabolites (such as acylcarnitines) and immune-inflammatory signals (such as NF-κB activation) (146, 147). For instance, while glomerular barrier damage is associated with dysregulated endothelial S1pR1 signaling (145) and certain gut-derived metabolites can restore intestinal barrier function via the AHR receptor (148), integrated models addressing cross-talk between barriers in DN are still missing.

This fragmented approach generates significant limitations in both mechanistic understanding and therapeutic development. Single-pathway analyses, while facilitating understanding of specific molecular events—such as brown adipose tissue-derived NRG4 suppressing podocyte apoptosis through the Akt-GSK3β pathway (149) —overlook the broader impact of cross-talk among metabolic, immune, and barrier pathways (150, 151). Consequently, therapies targeting isolated pathways, such as PI3K-Akt or HIF-1 signaling, often fail to achieve optimal efficacy due to neglect of feedback and interaction between pathways (152, 153). For example, while PFKFB3 contributes to glomerular barrier protection (145), its interactions with microbial metabolic profiles, including circulating acylcarnitine levels (139), remain absent from therapeutic considerations.

In summary, the current fragmented approach constrains both mechanistic insights and identification of effective therapeutic targets. Comprehensive, multi-dimensional studies integrating metabolism, immunity, and barrier biology are urgently needed to clarify DN pathogenesis and guide the development of more effective interventions.

### 5.3 Barriers to clinical translation

The clinical translation of GM-based interventions for DN faces significant hurdles, primarily arising from the disconnect between findings in animal models and human clinical realities, as well as the lack of longitudinal, stage-specific data in disease progression. These barriers substantially impede the transition from bench to bedside.

One core challenge lies in the differences between animal and human GM, which restrict the reproducibility and translatability of preclinical findings. The GM composition in common animal models—such as rodents—differs markedly from that of humans, resulting in inconsistencies in intervention efficacy. Mechanisms such as short-chain fatty acid metabolism or intestinal barrier repair, which often demonstrate efficacy in animal studies, may not be directly reproduced in clinical studies due to these interspecies differences (154, 155). This limitation raises concerns regarding the extrapolation of preclinical results to human disease contexts.

Additionally, there is a conspicuous lack of systematic, longitudinal data capturing the dynamic shifts of GM throughout the various stages of DN. Most current studies are cross-sectional in design, typically comparing diabetic patients to healthy controls, and fail to longitudinally track changes in GM across clinical stages—from microalbuminuria to established renal failure (156, 157). This gap obscures the understanding of how microbial communities evolve with disease progression and limits the identification of stage-specific therapeutic targets.

**Figure 3:**
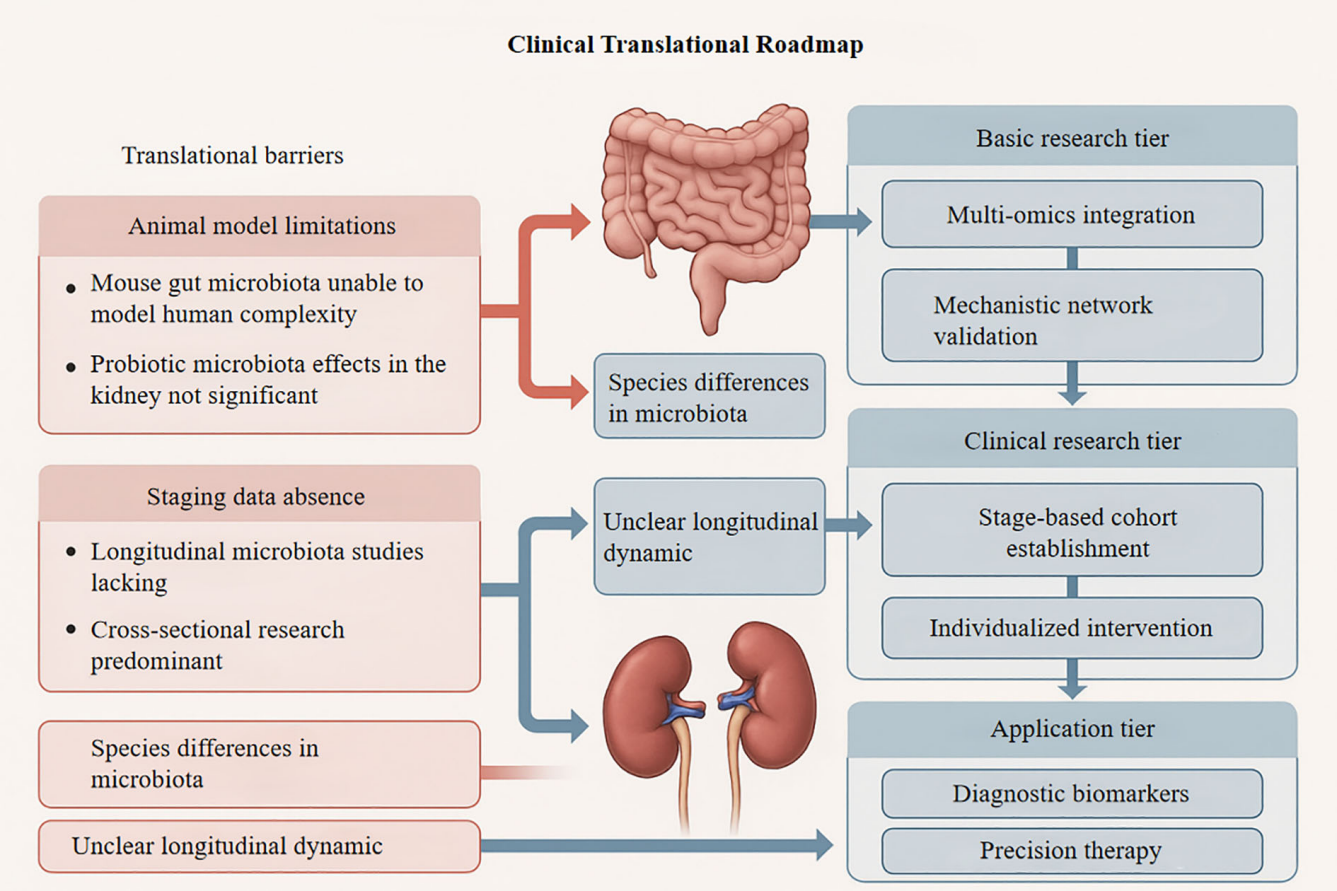
Roadmap for translating microbiota- kidney research into clinical applications, with key barriers identified. This schematic outlines a three-tiered framework for advancing gut-kidney axis research. Left panel highlights translational barriers (red boxes): (i) Animal model limitations (non-human microbiota complexity; limited probiotic efficacy in renal contexts), (ii) Staging data gaps (predominance of cross- sectional studies; longitudinal data scarcity), and (iii) Unresolved microbial dynamics (species-specific variations; undefined longitudinal trajectories). Center depicts gut- kidney anatomical targets. Right vertical axis details progressive research phases: Basic research (multi-omics integration; mechanistic network validation) → Clinical research (stage- stratified cohorts; individualized interventions) → Application (diagnostic biomarkers; precision therapies). Blue arrows denote barrier impact on translational stages. Kidney protection seen in animal models using probiotics, antibiotics, or fecal transplants often proves less effective in human trials. This is partly because animal models inadequately capture the complexity of the human gut- kidney axis and host-microbe co-evolution, making therapeutic targets identified in animals less reliable for clinical application.

The absence of robust, disease stage-oriented microbial evolution data hinders the development of tailored intervention strategies—for example, metabolic modulation in early-stage DN versus barrier restoration in advanced stages. Consequently, the true potential of personalized, microbiota-based therapies remains largely unrealized. The clinical translation workflow is summarized in **Figure 3**.

## 6 Innovation and future directions

With the growing recognition of the GM’s pivotal role in DN, innovative research is increasingly oriented toward integrating mechanistic insights, multi-omics approaches, and personalized interventions. The following subsections systematically summarize key innovations and emerging trends in this field.

### 6.1 Integration of gut–kidney axis mechanisms and targeted interventions

At the mechanistic level, recent studies have, for the first time, systematically linked gut dysbiosis in DN with targeted intervention strategies. Specifically, current literature has demonstrated that gut microbial imbalance exacerbates glomerulosclerosis and tubular injury through both metabolic products (e.g., uremic toxins) and immune-inflammatory pathways (e.g., activation of Toll-like receptors) (17, 44). On this basis, targeted interventions aimed at restoring the gut–kidney axis—such as probiotics and prebiotics—may modulate microbial metabolism (e.g., enhancing short-chain fatty acid synthesis) and improve renal injury biomarkers, thereby attenuating kidney damage (158, 159).

### 6.2 Multi-omics research paradigms across scales

Multi-omics approaches have ushered in a new framework for dissecting the mechanisms of GM in DN. Metagenomics can reveal shifts in microbial community structure among DN patients, such as alterations in the abundance of specific genera (23, 51). However, coupling with metabolomics is required to delineate the specific nephrotoxic effects of microbial metabolites like indoxyl sulfate (79). Moreover, metatranscriptomics allows for the identification of key microbe–host interaction pathways, including the regulation of inflammatory responses via the AhR pathway (18, 160), while single-cell techniques enable detailed analysis of intestinal immune cells (e.g., Th17/Treg) and their association with renal immune infiltration (160, 161). Collectively, integrated multi-omics approaches hold promise for elucidating cross-scale regulatory networks linking microbial structure, function, and host physiology (79, 162).

### 6.3 Stage-specific and precision intervention strategies

Strategic interventions must be tailored to distinct DN stages. In early DN, supplementation with short-chain fatty acid-producing bacteria (such as Butyricicoccus) or their metabolic products (such as butyrate) has the potential to improve insulin resistance and alleviate glomerular hyperfiltration (158, 159). In contrast, late-stage interventions require the elimination of toxin-producing bacteria (such as Escherichia species) and blockade of toxin absorption (44). Nonetheless, major obstacles remain, including inter-individual variability in response to microbiota-based therapies due to genetic and dietary factors, as well as incomplete evidence regarding long-term safety (35, 163).

### 6.4 Potential of advanced technologies

Frontier technologies offer new avenues for GM-targeted therapy. Phage therapy can specifically lyse pathogenic bacteria (such as endotoxin-producing Klebsiella), and animal models have confirmed its ability to reduce serum LPS levels and proteinuria (25). Meanwhile, engineered probiotic therapies—where symbiotic bacteria are modified to express therapeutic molecules such as antioxidant enzymes—demonstrate potential in locally mitigating renal oxidative stress (45, 159). Going forward, the development of synergistic systems (e.g., coordinated phage–engineered bacteria interventions) may enable dynamic modulation of gut microbial ecology (25).

In terms of clinical translation, future directions include stratified and personalized probiotic regimens based on enterotype (161, 162), incorporation of microbiota-derived biomarkers (such as fecal butyrate) as efficacy indicators (164), and the application of artificial intelligence models to integrate multi-omics data for predictive risk assessment of DN progression (162).

## 7 Conclusion

The gut-kidney axis plays a pivotal role in DN pathogenesis through distinct yet interconnected mechanisms. Gut dysbiosis drives DN progression via three pathways: metabolic dysfunction through loss of protective SCFAs and accumulation of nephrotoxic compounds like indoxyl sulfate; compromised intestinal barrier integrity leading to endotoxin translocation and systemic inflammation; and immune dysregulation affecting Th17/Treg balance and macrophage polarization. MR studies have validated specific microbial signatures associated with DN risk, including protective effects of Bacteroidota and detrimental impacts of reduced Akkermansia abundance.

Current therapeutic approaches demonstrate promise but require optimization. Probiotic supplementation and TCM interventions have shown benefits in improving renal function markers and restoring gut barrier integrity, yet standardization of protocols remains a critical challenge. Future research priorities include elucidating molecular mechanisms linking specific bacterial strains and their metabolites to renal pathology, integrating multi-omics approaches for patient stratification, and developing DN-specific microbial biomarkers for early diagnosis.

Advancing the field necessitates interdisciplinary collaboration between microbiome researchers, nephrologists, and nutrition scientists to develop personalized therapeutic strategies. Key challenges include addressing regional microbiota heterogeneity and the complexity of host-microbe interactions through large-scale prospective cohorts and mechanistically driven randomized controlled trials. As our understanding of the gut-kidney axis deepens, microbiota-targeted interventions represent a promising frontier for DN management, offering potential for both prevention and treatment through precision medicine approaches.
